# Interactions Between Nanoparticles and Tomato Plants: Influencing Host Physiology and the Tomato Leafminer’s Molecular Response

**DOI:** 10.3390/nano14221788

**Published:** 2024-11-07

**Authors:** Inzamam Ul Haq, Xiangyun Cai, Habib Ali, Muhammad Rehan Akhtar, Muhammad Adeel Ghafar, Moazam Hyder, Youming Hou

**Affiliations:** 1 State Key Laboratory of Ecological Pest Control for Fujian and Taiwan Crops, Key Laboratory of Biopesticides and Chemical Biology, MOE, College of Plant Protection, Fujian Agriculture and Forestry University, Fuzhou 350002, China; 000b370305@fafu.edu.cn (I.U.H.); 2220203003@fafu.edu.cn (X.C.); m.rehan@fafu.edu.cn (M.R.A.);; 2 Department of Agricultural Engineering, Khwaja Fareed University of Engineering and Information Technology, Rahim Yar Khan 64200, Punjab, Pakistan; habib.ali@kfueit.edu.pk

**Keywords:** agricultural nanotechnology, nanoparticles, pest management, plant resistance, tomato leafminer

## Abstract

Tomatoes are a crucial global crop, impacting economies and livelihoods worldwide. However, pests like the tomato leafminer (*Tuta absoluta*) significantly reduce their yield potential. Nanoparticles come as a solution to this context, promising innovative strategies for the protection of plants from pest infestation and management. Nanoparticles have shown great potential to improve tomato plant resistance against pests and diseases because of their unique properties. They enhance plant physiological processes like photosynthesis and nutrient uptake while activating defense-related molecular pathways. Nanoparticles also directly impact the life cycle and behavioral patterns of pests such as the tomato leafminer, reducing their destructive nature. The dual benefits of nanoparticles for enhancing plants’ health and managing pests effectively provide a two-way innovative approach in agriculture. Gains made with such technology not only include increasing crop productivity and reducing crop losses but also reducing the heavy dependence on chemical pesticides, many of which have been attributed to environmental hazards. The current study illustrates the broader implications of nanoparticle use in agriculture, which is a sustainable pathway to increase crop resilience and productivity while reducing the impact of pests. Such novel approaches underline the need for continued interdisciplinary research to exploit the potential of nanotechnology in sustainable agricultural practices fully.

## 1. Introduction

Tomatoes (*Solanum lycopersicum*) represent not only an essential part of the culinary traditions of most countries but also a cultigen that has an enormous effect on the world agriculture market and remains one of the critical sources for the nutritive value of millions [1]. Tomatoes remain one of the common vegetables consumed across the world, contributing to the livelihoods of farmers, especially in countries like China, India, and the United States, which rank top in their production [2]. The global tomato industry holds significant economic value, especially in high-producing countries [3].

Tomatoes provide essential vitamins, minerals, and antioxidants beneficial for health [4,5]. Tomatoes’ versatility in culinary application—whether fresh in salads or cooked in dishes—solidifies them as one of the world’s favorite culinary and nutritional components [6].

However, the tomato leafminer *Tuta absoluta* (Meyrick) poses a severe and costly threat, capable of devastating crops and affecting yields globally. *T. absoluta* is an invasive and devastating pest of solanaceous crops that has spread globally [7]. It originated in South America, and its recent invasion and trailing across Europe, Africa, and some parts of Asia have caused a severe threat to the tomato field since it has high fecundity and aggressive feeding behavior [8,9]. *T. absoluta*’s biology features a high reproduction rate and capability to cycle from egg to adult in under a month under most conditions, with as many as 12 annual generations attainable in warmer climates

This pest has four stages in its life cycle: egg, larva, pupa, and adult [10]. The larvae cause damage, burrowing into tomato leaves, stems and fruits, producing visible mines or pupal dots, and ultimately weakening plant structure and the aesthetic of the crop [11]. The function not only results in direct yield losses but also lowers the food value of the crop, with important implications in terms of farmer income and market supply [12].

The effect of *T. absoluta* on the economy is substantial, such that infestations can even result in up to 100% crop loss if not controlled. Historically, pest management has relied heavily on chemical pesticides due to their effectiveness in immediate pest control. However, concerns over environmental impact, pesticide resistance, and residue on crops have necessitated alternative approaches, such as biological control methods. These methods, which leverage natural predators and parasitoids, offer eco-friendly solutions but face limitations in consistency and climate dependency [11,13].

Conventional control methods, such as chemical pesticides, face challenges like environmental impact, pesticide resistance, and economic costs. The main challenges faced by these control methods include low effectiveness and ecological effects. Chemical control is effective in the beginning but experiences the problem of pesticide resistance [14]. Repeated application of chemical pesticides has led to increased resistance in *T. absoluta* populations and reduces the long-term viability of this method [15]. Additionally, the excessive use of chemical pesticides can leave residues in the produce, which pose a health risk to the consumers and will limit export opportunities due to the imposition of severe international residue limits [16].

The biological control methods comprising natural predators and parasitoids of *T. absoluta* offer a more environmentally friendly option [17]. Such methods typically require adequately managed conditions for their success, such as maintaining adequate populations of the natural enemies and suitable environmental conditions, which can be challenging to obtain and keep on extensive farmlands [18]. Moreover, climatic conditions are noted to interfere with biological agents’ effectiveness, restricting their usage in areas with less favorable climates [19].

Both strategies also result in environmental degradation: the chemical methods act on non-target species and biodiversity, while the other method may cause an unintended impact on the ecosystem [20]. These cons, therefore, warrant the development of innovative and more sustainable pest management strategies for crop security and environmental health over the long term.

Given the limitations of chemical and biological control methods, advanced approaches like nanotechnology have emerged to address pest management challenges more sustainably. Synthesized nanoparticles, including silver, zinc oxide, and silica nanoparticles, have garnered attention for advancing sustainable agricultural practices. Engineered to possess unique physicochemical properties, such as increased surface area and reactivity, these nanoparticles enable targeted applications in plant protection and growth enhancement [21]. Thus, agriculture applications have also utilized nanoparticles’ unique properties concerning size and surface area to allow tailoring for precise functionalities or tasks. For example, it may be possible to design nanoparticles that release pesticides in a controlled manner at doses low enough to minimize environmental exposure yet still practical, reducing the number of applications. This controlled release also assures the continuation of the active principle available for a longer duration against insects like *T. absoluta* and can lead to more successful pest control [22].

Other nanoparticles exhibit inherent pesticidal activity. For example, silver nanoparticles are a potential antimicrobial and insecticidal agent because they effectively interfere with the growth and reproductive cycles of major pests without using other chemical agents [23]. Such bioactivity is crucial because it reduces reliance on synthetic chemicals, which are mostly considered resistant and contaminating the environment.

Unlike traditional chemical applications, nanoparticles can be tailored for controlled release and precision targeting, reducing environmental impact and addressing some of the key challenges in conventional pest control. This approach represents a significant evolution in pest management technology, leveraging nano-scale properties to bolster plant resilience and pest deterrence. In addition, the nanoparticles may enhance the plant’s natural defense capacity against pests. A few studies indicate that some nanoparticles can improve plants’ immune systems to the point where they can resist pests and diseases through priming [24]. This leads, in return, to a quick and robust activation of defensive responses once the plant is attacked by pests [25]. Incorporating nanotechnology into pest management in agriculture assures effectiveness in controlling the population and agrees with the modern need for sustainability in agriculture. In this light, this innovative approach might redefine pest control strategies with a realistic alternative view against many conventional methods, which are usually unsustainable and increasingly ineffective.

## 2. Overview of Nanoparticles in Agriculture

### 2.1. Types and Properties 

Nanoparticles (NPs) are generally defined as particles with dimensions between 1 and 100 nanometers, a size range that enables unique physical and chemical properties. Based on their size, NPs can be further classified into three sub-categories: ultrafine (1–10 nm), nano-scale (10–100 nm), and larger colloidal particles that may still exhibit nano-properties but exceed 100 nm in at least one dimension. The smaller the particles, the greater the surface area to volume ratio, enhancing reactivity and making them more effective for targeted applications in plants [26,27]. Nanoparticles can also be classified by their origin, including natural, incidental, and engineered nanoparticles. Natural nanoparticles occur in the environment and include particles like volcanic ash or mineral oxides. Incidental nanoparticles are by-products of combustion and industrial processes. Engineered nanoparticles, which are intentionally designed and synthesized, represent the majority of agricultural applications and include materials like silver, zinc oxide, and silica nanoparticles [28,29]. Each type of NP origin affects its composition, properties, and application potential in agriculture [30,31]. Among these, silver nanoparticles have gained much interest due to their antimicrobial property associated with the released silver ions. These may interfere with DNA replication and disturb microbial cell membranes [32]. Therefore, silver nanoparticles are effective against various plant pathogens and pests. Moreover, the small size with a large specific surface area of those nanoparticles allows easy uptake and rapid distribution within plant tissues to enhance protective effects [32].

The most widely used type of nanoparticle in agriculture is nano zinc oxide. These nanoparticles are mostly sought after for their contribution to the plant’s growth and tolerance [33]. Zinc is an essential microelement that activates plant enzymes and protein synthesis. Nanoparticles of zinc oxides provide bio-available forms of zinc, which can more readily be available to plants than conventional sources. This higher bioavailability will also increase the crop’s growth, yield, and disease resistance [34]. Silica nanoparticles further benefit plants by strengthening their cell walls and enhancing plant tolerance to biotic and abiotic stresses [35]. Silica is not only incorporated into the plant structure to have a role in modulating the physiological mechanisms of the plant but also confers improved resistance against pests through the silica effect that makes the plant tissue less palatable or penetrable by the pests [36,37].

Copper nanoparticles (CuNPs) possess strong antimicrobial properties, making them effective in reducing bacterial and fungal diseases in crops. Their unique redox activity disrupts microbial cell walls, providing a protective effect against pathogenic attacks [38]. However, at higher concentrations, CuNPs can cause phytotoxicity and may lead to bioaccumulation in edible parts, posing potential risks to human health if not carefully managed [39]. Titanium dioxide nanoparticles (TiO_2_ NPs) are widely used for their ability to enhance photosynthetic efficiency by improving light absorption. This property makes TiO_2_ NPs beneficial for increasing crop productivity, especially under suboptimal light conditions. Furthermore, TiO_2_ NPs have been shown to improve stress tolerance in plants [40]. However, concerns remain regarding their persistence in the environment and potential impacts on soil microbiota when applied in high doses [41]. Iron oxide nanoparticles (Fe_2_O_3_ NPs) play a crucial role in enhancing nutrient availability, particularly for iron-deficient soils. They increase iron bioavailability in plants, promoting chlorophyll production and overall plant vigor. Additionally, Fe_2_O_3_ NPs have been studied for their role in reducing pest-induced stress [42]. Nevertheless, at excessive concentrations, they may interfere with root development and impact nutrient balance [43].

While each type of nanoparticle offers unique benefits—such as CuNPs for pathogen resistance, TiO_2_ NPs for photosynthesis enhancement, and Fe_2_O_3_ NPs for nutrient supplementation—they also present challenges. These include the risk of phytotoxicity, environmental persistence, and potential impacts on non-target organisms. Careful consideration of nanoparticle type, application rate, and ecological factors is therefore essential to maximize agricultural benefits while minimizing adverse effects.

### 2.2. Plant Interaction 

**Figure 1:**
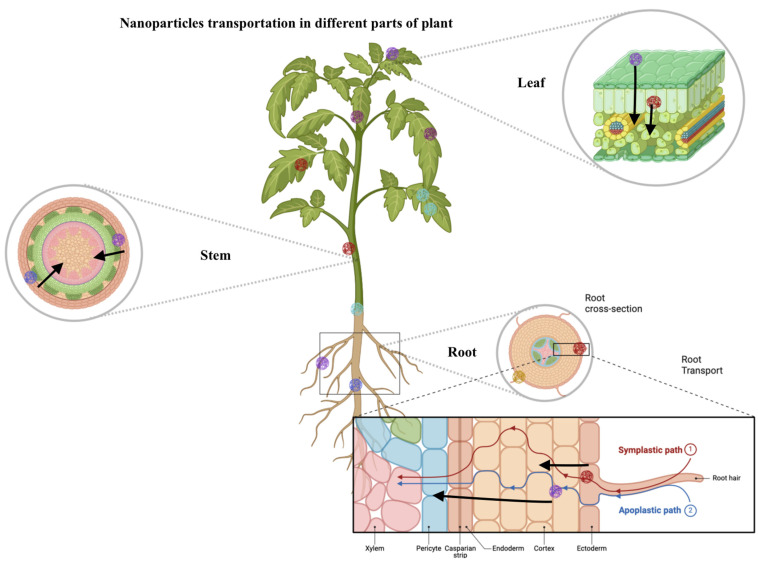
Uptake and translocation of nanoparticles in tomato plants. This figure illustrates the absorption and distribution pathways of nanoparticles in tomato plants. It shows how nanoparticles penetrate through stomata openings or the cuticle on the leaf surface, move within the leaf tissue, and are translocated to other plant parts via the xylem. The figure highlights the mechanisms by which nanoparticles improve nutrient uptake, enhance plant resistance, and contribute to targeted delivery of agrochemicals.

The interaction of nanoparticles with plant systems is complex, involving processes of absorption, uptake, and translocation, which are principal in controlling the bioefficacy of these nanoparticles in agricultural applications [44,45]. Nanoparticles can enter the plant through the roots, leaves, or stems, depending on the mode of application: soil amendment, foliar spray, or stem injection (Figure 1). Owing to their small size and characteristic surface properties, the nanoparticles are transferred to various plant barriers and channels once they get taken up into the plant. The uptake of nanoparticles in roots is conducted by the exact mechanism with which nutrients are taken up; it involves moving them from the soil and into the root cell. From these cells, nanoparticles may be translocated to other parts of the plant via the xylem [46]. Thus, the process is driven by the plant’s transpiration stream, while the nanoparticles, on their part, are distributed in the plant, to the stems, leaves, and sometimes even to the fruits and seeds [47].

Foliar application occurs through the absorption of the nanoparticles on stomata openings or through the cuticle on the leaf surface. The nanoparticles are taken in either passively because of their nano-size or actively, possibly facilitated by changes they induce in the permeability of cellular membranes [48]. Once inside the leaf tissue, these nanoparticles can move either cell to cell or get loaded into the phloem for transport to other plant parts [49]. Absorption and distribution of nanoparticles in plant systems are a prerequisite for targeted delivery of agrochemicals, improved nutrient use, and enhancing plant resistance mechanisms against stresses. However, the effectiveness of these processes can vary widely based on the type of nanoparticle, its size, surface charge, and coating, as well as the species of plant and the environmental conditions [50].

### 2.3. General Plant Benefits 

Nanoparticles offer many general benefits for plants, such as significant increases in growth rate and stress tolerance. Evidence also indicates that nanoparticles modulate the various aspects of plant physiological and biochemical processes towards increased photosynthetic activity and nutrient uptake, subsequently enhancing growth rates [51]. For instance, titanium dioxide nanoparticles have been proven to enhance photosynthesis in spinach plants to improve biomass production [52]. This enhancement is attributed to the property of nanoparticles to absorb and scatter light, which in turn increases the efficiency of photosynthesis.

The improvement in the nutritional status of plants through nanoparticles has been found to enhance nutrient uptake by zinc oxide and iron oxide. These nanoparticles help transport and assimilate essential plant nutrients such as zinc and iron, which are essential for plants’ growth and development [25]. It also increases the plants’ growth rate, thereby giving out improved plant products with more nutritious attributes for human health values. The other known advantages of using these nanoparticles are that they make plants more tolerant to several kinds of stresses they have to face, such as drought, salinity, and pathogen attack. For example, it has been proven that silver nanoparticles enhance maize drought tolerance by the regulated expression of osmotic-stress proteins and inducting the activity of antioxidant enzymes in the plant. Similarly, silicon nanoparticles have successfully elevated tolerance against salinity in tomato plants by upregulating cellular defense and balancing ion homeostasis [53].

Recent research emphasizes the importance of understanding the molecular interactions between nanoparticles and plants. According to Singh, et al. [54], nanoparticles can modulate gene expression related to stress responses, effectively priming plants to withstand biotic and abiotic stresses more robustly. Additionally, Francis, et al. [55] discuss how metal nanoparticles interact with cellular pathways, affecting nutrient uptake and metabolic functions, which has implications for sustainable crop improvement and agricultural productivity.

### 2.4. Comparative Effectiveness of Nanoparticles in Agriculture

Each nanoparticle type discussed in this study exhibits unique advantages for enhancing plant resilience and pest management, contributing to the broader goals of sustainable agriculture. Silver nanoparticles (AgNPs), renowned for their antimicrobial capabilities, disrupt critical biological pathways in pests—such as oxidative stress and detoxification processes—making them especially potent for pest suppression [56,57]. Zinc oxide nanoparticles (ZnO NPs), by contrast, play a significant role in improving plant growth through enhanced nutrient uptake and enzymatic support for photosynthesis, which collectively strengthens plant health and resilience against environmental stressors [33,34]. Similarly, silica nanoparticles (SiO_2_ NPs) fortify plant cell walls and activate jasmonic acid signaling pathways, thereby decreasing plant palatability to herbivorous pests and acting as an effective deterrent [36]. Copper nanoparticles (Cu NPs) further exhibit strong antimicrobial properties and disrupt reproductive pathways in pest larvae and eggs, supporting effective population control measures [58]. Lastly, titanium dioxide nanoparticles (TiO_2_ NPs) contribute to photosynthetic efficiency and biomass production by improving light absorption, offering benefits for crop productivity in varied environmental contexts [40,52]. Collectively, these nanoparticles provide a comprehensive set of tools for agricultural sustainability, from direct pest suppression to the enhancement of plant defense mechanisms, thus aligning with the increasing need for innovative and environmentally sustainable pest management practices.

## 3. Impact of Nanoparticles on Tomato Plant Physiology

### 3.1. Physiological Enhancements

These nanoparticles can significantly improve the physiological functioning of tomato plants, including photosynthesis and nutrient uptake. Evidence suggests that some nanoparticles, such as titanium dioxide and zinc oxide, enhance the photosynthetic capacity of a plant. For example, it has been shown that titanium dioxide nanoparticles enhance the ability for light absorption and hence increase chlorophyll photoreactivity, contributing to the improvement of photosynthesis and biomass in tomato plants [40]. This photosynthetic efficiency improvement significantly increases crop productivity, especially under limited light conditions.

Nanoparticles such as zinc oxide and iron oxide participate in the uptake of nutrients. They offer highly essential trace elements in a bioavailable form, quickly and efficiently uptaken into tomato plants [59]. For example, this can be represented by applying zinc oxide nanoparticles, which enhance the concentration of tissues in plants, serving as one of the most essential elements for enzyme activation and protein synthesis. This improved micronutrient status will enhance better growth and overall health and vigor, enabling the plant to be better equipped to resist stresses and diseases. In addition, the nanoparticles also alter root architecture, leading to a more extensive root system, hence increasing water and nutrient uptake from the soil by the plant [60]. This betterment of root development greatly aids in situations where the soil is nutrient-poor and might contribute much to sustainable agricultural practice by reducing dependence on chemical fertilizers.

### 3.2. Pathway Activation 

**Figure 2:**
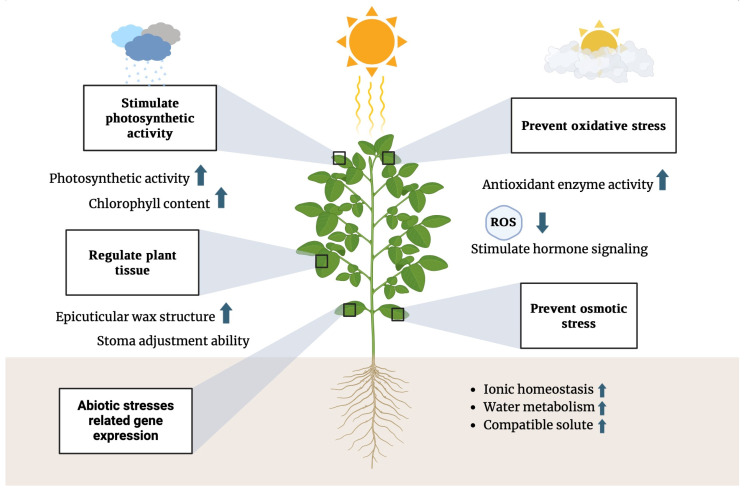
Activation of plant defense mechanisms by nanoparticles. This figure depicts the various defense pathways activated in tomato plants in response to nanoparticle exposure. Blue arrows indicate the upregulation or downregulation of specific signaling pathways, including oxidative stress response, salicylic acid (SA) signaling, and jasmonic acid (JA) pathways, as well as systemic acquired resistance. These regulatory changes strengthen the plant’s immune response, enhancing defense against biotic and abiotic stresses.

A wide variety of molecular pathways can be triggered in tomato plants in response to the application of nanoparticles, for example, those related to defense, particularly oxidative stress and hormonal signaling (Figure 2). These nanoparticles cause a stress reaction in the plant similar to that of a pathogen’s attack and induce the plant to reamplify its defense mechanisms. For example, it has been demonstrated that silver nanoparticles can induce reactive oxygen species (ROS) in plant tissue; these are harmful but are believed to be a necessary part of the signaling process that switches on the expression of defense genes [61]. This oxidative burst is a typical response observed during pathogen infection and is crucial for inducing other defense pathways.

In addition, apart from oxidative stress pathways, nanoparticles can act on hormonal signaling pathways that also participate in plant defense, such as those mediated by salicylic acid (SA) and jasmonic acid (JA). These hormones are vital in the plant defense response to biotic and abiotic stresses. As an illustration, when there is an enhancement in the salicylic acid pathway, there is systemic acquired resistance by zinc oxide nanoparticles—one of the mechanisms for “whole-plant” resistance to pathogens [62]. It was found that silica nanoparticles activated the jasmonic acid pathway in defense against insects and necrotrophic pathogens [63].

### 3.3. Defense Mechanisms

The unique properties of nanoparticles enhance immune responses and induce plant resistance mechanisms. Their small size enables penetration and interaction with cellular components to activate several defense pathways. For instance, nanoparticles may act as elicitors stimulating phytoalexin production—an antimicrobial compound produced by plants in response to the attack of pathogens. This response helps prevent pathogen activity and primes the plant to respond more vigorously to future attacks.

Furthermore, the nanoparticles can give systemic resistance in plants. This means that the treated part of the plant sends a signal about the threat to other parts, therefore helping to build up the immunity of the whole plant. For instance, it is stated that silica nanoparticles induce systemic acquired resistance to plants through increasing the accumulation of signaling molecules, among them salicylic acid, which plays essential roles in plant defense against pathogens (Table 1). Such a type of resistance is precious because it protects against a great range of pathogens. It has also been reported that applying silver and copper nanoparticles could activate enzyme-based defense systems in a plant. These include peroxidases, superoxide dismutases, and catalases, which have already been associated with detoxifying reactive oxygen species during stress responses, protecting cells from oxidative damage, and helping the plant cope with environmental stresses better [43].

## 4. Direct Effects of Nanoparticles on the Tomato Leafminer

### 4.1. Lifecycle Changes

Nanoparticles have been tested for their effects in different life cycle stages of several agricultural pests, including the tomato leafminer *T. absoluta*. The work shows that nanoparticles influence the growth and development of the same pests, causing lowered survival and disturbing their reproduction cycles. For example, silver nanoparticles are effective at changing the developmental status of insects by interfering with larval and pupal stages of insects, thus mainly being able to reduce the population of a succeeding generation [43].

Nanoparticles affect insect pests like *T. absoluta* through multiple mechanisms, including the generation of oxidative stress, cellular disorganization, and disruption of the molting process. Due to their nano-scale size, these particles can penetrate the insect cuticle and accumulate within tissues, thereby interfering with essential biochemical and physiological pathways [67]. Studies have shown that nanomaterials, such as silver and zinc oxide nanoparticles, induce oxidative stress by generating reactive oxygen species (ROS), leading to damage in proteins, lipids, and DNA within insect cells [68,69]. Such oxidative damage has been observed to impact reproductive and developmental processes in insects, supporting their potential as an effective pest control strategy [70].

**Figure 3:**
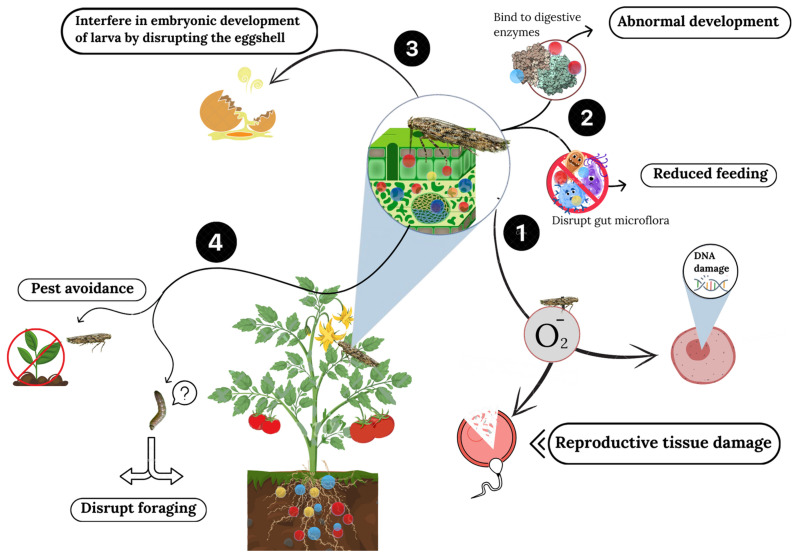
Behavioral and physiological effects of nanoparticles on the tomato leafminer. This figure illustrates the direct and indirect effects of nanoparticle exposure on the behavior and physiology of the tomato leafminer. It shows significant alterations in food intake, movement patterns, and overall activity, along with nutrient metabolism and digestion disruptions. The figure also highlights the impact of nanoparticles on the survival and reproduction potential of the pest, contributing to its management.

The nanoparticles can interfere with the absorption of nutrients in insects by bonding to digestive enzymes or disrupting gut microflora, which participates in the digestion process of many pests (Figure 3). This will, in turn, not only lead to interference with the growth and development of the pests but also decrease their feed efficiency on treated plants, lowering their overall virulence [71].

### 4.2. Reproductive Effects 

Nanoparticles have shown potential impacts on the reproductive capabilities of agricultural pests, with *T. absoluta* being no exception. Several studies have investigated exposure to nanoparticles, metal-based nanoparticles such as silver and copper, and their consequences on the fertility and fecundity of these insects. Due to these sub-lethal effects caused by the nanoparticles, they can cause a drastic reduction in reproduction, posing a challenge to any such reproductive characteristic of an insect species [58]. For example, exposure to silver nanoparticles leads to malformations in reproductive organs and eggs, decreasing the egg-laying capacity and increasing larval and pupal mortality—all of which contribute to reduced population growth [64]. Postulated mechanisms behind these include the induction of oxidative stress that may cause damage in the reproductive tissues and the potential disruption of hormone regulation, which is necessary for normal processes involved with insect reproduction.

Copper nanoparticles interfere with the integrity of the eggshell and impair the embryonic development of larvae contained within the egg, affecting the hatchability of insect eggs [65]. Such an effect will not only directly influence the current generation but potentially significantly impact population dynamics over the years.

### 4.3. Behavioral Changes

Exposure to nanoparticles, particularly in treated plants, has shown significant impacts on the behavior of pests like *Tuta absoluta*. These impacts include reduced food intake, altered movement patterns, and diminished activity levels, affecting survival and reproduction potential. For example, Marouf and Allah [72] found that nanosilver impacts pest behavior by reducing feeding rates and movement. Additionally, Wang, et al. [73] demonstrated how nanocarrier systems targeting *T. absoluta* lead to disrupted feeding and reproduction patterns. Research has shown that upon encountering nanoparticle-treated plants, leafminers and other related insects have suppressed their feeding activity [74]. This is perhaps associated with the changed chemical composition of plant tissues, such as nanoparticles in the tissues themselves, or changes in the plant’s chemical defenses mediated by the exposure to nanoparticles [75]. For instance, silver nanoparticles have been reported to change the surface chemistry of plant leaves to render them less palatable to pests, and therefore, consumption and, consequently, damage to the plant are reduced [57].

Besides that, it might even impact pests’ locomotion and orientation behaviors, which are essential for survival and locating a suitable mate [76]. Previous studies on nanoparticles based on copper showed disturbances in their standard patterns of movement, probably because of neurological impairment or depletion of energy due to the toxic effects of the nanoparticles [77]. Such disturbances can leave pests unable to find their food resources or escape bad situations, so their chances of survival are severely limited.

## 5. Indirect Effects via Altered Host Plant Physiology

### 5.1. Nutrient Metabolism Disruption

**Figure 4:**
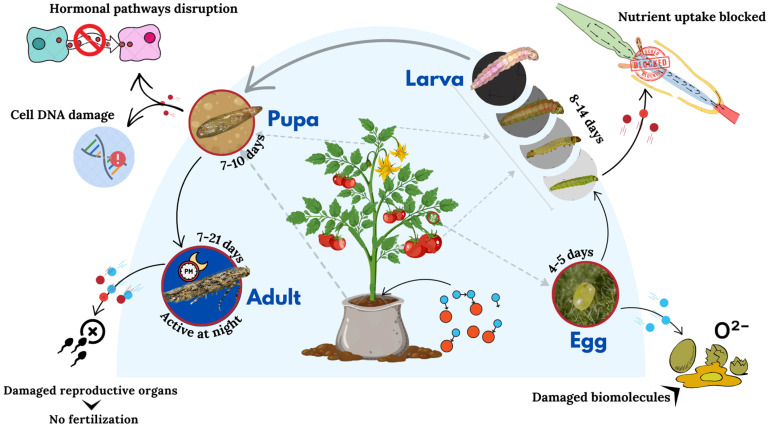
Transcriptomic and proteomic changes in the tomato leafminer induced by nanoparticles. This figure presents the molecular changes observed in the tomato leafminer after feeding on nanoparticle-treated plants. It shows altered gene expression profiles related to detoxification, stress response, reproduction, and proteomic changes affecting critical biological processes. The figure underscores the potential of nanoparticles to disrupt the normal physiological functions of pests, enhancing their susceptibility to plant defenses.

Applying nanoparticles to the plants exposes them to physiological changes affecting pests’ nutrient metabolism, such as *T. absoluta*. Nutrient metabolism in an insect may be affected by alternative nutrition, which could be due to an abnormal metabolite or severe suppression of precursor substances in the normal pathway of nutrient transformation (Figure 4). When feeding on such nanoparticle-treated plants, these disturbances can manifest in significantly disturbed normal nutrient metabolism processes in the pests. It was found that, for instance, silica and zinc oxide nanoparticles may modify the nutrient content and distribution in plant tissue [78]. These effects could render the nutrients less available to, or less digestible by, the pests, with negative consequences on their growth and development. As an example, silica nanoparticles deposit within the cell walls of plant tissues, and it was demonstrated that they increase structural barriers, which can slow down the digestibility of leafminers while feeding on a plant by reducing their digestive enzymes’ capability to extract nutrients efficiently [79].

In addition, nanoparticles can stimulate the production of secondary metabolites in the plant, some of which may be toxic or act as repellents. Such would not only influence the palatability of the plant tissues but may bring about a metabolic disruption in the pests feeding on such tissues. For instance, plants treated with copper nanoparticles showed higher contents of phenolics and flavonoids, leading to an interruption in the metabolic pathways of insects upon feeding on them [66].

### 5.2. Transcriptomic Changes

The studies investigating the transcriptomic changes in pests such as the tomato leafminer, *T. absoluta*, after feeding on nanoparticle-treated plants have provided important new insights into how these particles alter gene expression in insects. Exposure to nanoparticles can induce molecular changes in pests that could modify some of the most critical genes in their metabolism, stress response, or reproduction. For example, it has been shown by RNA sequencing research that feeding with the silver nanoparticles-treated plant causes severe changes in the gene expression profiles of *T. absoluta*. These include genes associated with detoxification processes by metabolizing secondary metabolites and agrochemicals released by plants, such as cytochrome P450 enzymes. This may decrease the efficacy of detoxification and increase susceptibility to plant defenses and other insecticides [56].

In addition, other studies have reported gene expression changes involved in hormone signaling pathways responsible for regulating development and reproduction in leafminers. For instance, regulation in genes controlling the level of juvenile hormone and ecdysteroid, which are crucial in insects for events such as molting and maturation, shows downregulation with nanoparticle exposure. This may disrupt normal development processes, resulting in stunting or malformation in growth [80].

### 5.3. Proteomic Alterations

Most works have focused on proteomic changes by pests like *T. absoluta* when feeding by nanoparticle-treated plants. Such studies suggest that interactions with nanoparticles may significantly alter the protein expression profile, compromising critical biological processes or pathways necessary for these pests to survive and reproduce.

Studies have shown that proteins related to stress, metabolic processes, and detoxification are the most affected. For example, exposure to silver nanoparticles has resulted in the overexpression of heat shock proteins and other stress-related proteins in insects. These are essential proteins for facilitating the ability of organisms to cope with biotic and abiotic stresses; therefore, nanoparticles could induce a stress response in the leafminer [81]. In addition, the expression of genes related to energy metabolism, such as those associated with glycolysis and the production of mitochondrial energy, has been reported as being altered. This would impact the energy balance within the pest and hence the organism’s fitness, making it less vigorous with low reproduction [82].

Proteins associated with the insect detoxification system, such as various cytochrome P450s, glutathione S-transferases, and carboxylesterases, were found to change in expression upon nanoparticle exposure. These changes could become debilitating to the insect in its detoxifying ability, with xenobiotics, and in dealing with oxidative stress (Table 2). They could increase its susceptibility to other pesticides and environmental stressors [83].

## 6. Environmental Considerations

Nanoparticles in agriculture harbors critical environmental and regulatory concerns about safety and long-term effects. The ecological risks of nanoparticles arise from their novel properties, which—though beneficial in targeted applications—can lead to unforeseen interactions with environmental systems [26]. For instance, the ability of nanoparticles to quickly move within the soil and water systems would lead them to enter the aquatic environment and become a risk factor to the non-target species. It has been shown that specific nanoparticles (silver nanoparticles, mainly) are toxic to aquatic organisms and can also interfere with microbial populations, which play a significant role in nutrient recycling and, thus, in ecosystem function [84].

Nanoparticles can accumulate in soil, where they might modify the physical and chemical properties of the soil environment. Research findings have shown them to harm soil microbiota, depending on their composition and concentration, because they reduce microbial diversity and activity. This might, in turn, impact soil fertility and plant health in a way that would, over time, seriously disrupt the productive capacity of agricultural land over the long run [85]. For instance, it has been indicated that the very high concentration of zinc oxide nanoparticles was phytotoxic and affected the growth of plants and microbial populations in the soil [86].

### 6.1. Risk Assessment for Human and Animal Health

While nanomaterials present innovative solutions for crop protection and productivity enhancement, their application in agriculture necessitates careful consideration of potential health risks for humans and animals. Due to their small size and high surface reactivity, nanoparticles can be absorbed by plant tissues and accumulate in edible parts, raising concerns about the ingestion of nanomaterials by consumers. Studies suggest that metal nanoparticles, including silver (AgNPs) and zinc oxide (ZnO NPs), may persist in plant tissues and be transferred to higher trophic levels through food consumption, thus posing risks if they accumulate to toxic levels [43,78].

Potential health risks associated with nanomaterials are linked primarily to their ability to generate reactive oxygen species (ROS), which can damage cellular structures in human and animal cells, leading to oxidative stress and inflammation. Studies on AgNPs, for instance, indicate that high exposure levels can lead to cytotoxicity and disrupt metabolic functions in mammalian cells, raising concerns for both direct and indirect ingestion through food chains [81]. Zinc oxide nanoparticles, although beneficial for plant growth, may also pose risks if not applied within safe concentration limits, as they can impact gut health and immune responses when ingested in significant quantities [86].

To mitigate these risks, regulatory bodies, including the European Food Safety Authority (EFSA) and the US Environmental Protection Agency (EPA), have established preliminary guidelines for nanomaterial use in agriculture. These guidelines recommend thorough risk assessments based on nanoparticle concentration, plant uptake, and human exposure estimates. Moreover, recent research highlights the need for a comprehensive understanding of nanoparticle behavior in agricultural ecosystems and food products. Risk assessments typically involve evaluating nanoparticle bioaccumulation, toxicity thresholds, and long-term effects on human and animal health [87]. Developing standardized protocols for safe application and monitoring is essential to maximize the benefits of nanotechnology while protecting consumers and the environment.

### 6.2. Concentration Analysis and Control of Nanomaterials in Agricultural Crops

Accurate monitoring and control of nanomaterial concentrations in plants are essential to ensure that agricultural products remain safe for consumption. Due to the unique properties of nanoparticles, including their small size and high reactivity, they can accumulate in different plant tissues, including leaves, stems, roots, and fruits. However, precise methods are required to measure these concentrations at various stages of plant growth, as accumulation rates and distribution patterns can vary significantly depending on factors like plant species, nanoparticle type, and environmental conditions [43]. Several analytical techniques are currently employed to detect and quantify nanomaterials in plant tissues. Inductively Coupled Plasma Mass Spectrometry (ICP-MS) is a widely used method for measuring metal nanoparticles, such as silver and zinc oxide, due to its sensitivity and accuracy at trace levels. Additionally, techniques like Scanning Electron Microscopy (SEM) coupled with energy-dispersive X-ray Spectroscopy (EDS) allow for the visualization and elemental analysis of nanoparticles within specific plant tissues, providing insights into their localization and concentration [78,88].

Temporal monitoring is also critical. Regular analysis of nanoparticle concentrations during key growth phases, from early vegetative stages to harvest, is recommended to prevent excessive accumulation in edible plant parts. Studies indicate that nanoparticle uptake and translocation can be influenced by plant physiology and environmental factors, making it necessary to adjust nanomaterial application rates accordingly. Recent advancements in nano-sensor technology may offer in situ monitoring capabilities, enabling real-time concentration measurements directly in the field, which would support more precise control of nanomaterial levels in crops [89]. To minimize potential risks, guidelines from regulatory agencies advocate for establishing safety thresholds based on nanomaterial type and intended crop use. These guidelines emphasize the importance of balancing effective pest control or growth enhancement with safe concentration limits for human consumption. Developing standardized protocols for application and post-application monitoring will be essential for future nanomaterial use in agriculture, ensuring that concentrations in harvested products meet safety requirements [87]. 

## 7. Future Perspectives and Research Needs

### 7.1. Key Research Gaps and Future Directions

Among the areas requiring further research is establishing validated, reliable protocols for thoroughly characterizing nanoparticles in environmentally relevant, complex matrices. It will include the detection and quantification of NPs within soil and water systems and their fate under diverse environmental conditions. Such methods will be crucial in regulatory monitoring and compliance with safety standards in ecological systems. The last is a pressing need for interdisciplinary research that combines nanotechnology with plant and soil sciences, ecology, toxicology, and environmental science. This will provide an overview of how nanoparticles could be designed and applied successfully to agricultural safety with minimal adverse environmental impacts.

### 7.2. Broad Applications

Nanoparticles can provide a broad range of possible applications in controlling most agricultural pests and diseases by upping the ante on existing pest management strategies for various crops. Nanoparticles can be designed to specifically target pests and diseases with high efficacy while minimally affecting non-target species and decreasing the usual environmental footprint that is associated with traditional chemical pesticide applications. One example would be to develop nanoparticles containing fungicides or bactericides released in the presence of specific fungal or bacterial pathogens. The effectiveness and duration of treatment improve while the number of applications decreases.

Additionally, the nanoparticles can be functionalized to adhere to pests’ surfaces or even inside their digestive systems, further disturbing the pest’s physiological processes in a way that is impossible with other treatments. This strategy has indeed been investigated successfully with pests such as aphids and root-knot nematodes, which have been reported for interfering with feeding and reproduction processes, hence marking a significantly remarkable reduction of the pest population from the literature [90]. Besides direct antibacterial and antifungal activity, nanoparticles in plant disease management often elevate the plant’s innate immune responses, defined as ‘priming’. For example, silica nanoparticles have been demonstrated to induce systemic resistance in plants against various diseases by activating several signaling pathways related to plant defense [91].

### 7.3. Technological Advancements 

Future technological development will significantly advance the nanotechnological integration within sustainable farming practices, promising to increase agricultural productivity while minimizing environmental impacts. One of the most promising development areas is the creation of intelligent delivery systems that can release agrochemicals in response to environmental triggers such as changes in pH, temperature, or specific plant enzymes [92]. This allows for greater use efficiency of pesticides and nutrients through which this reduction of runoff and the associated potential for environmental contamination takes place. Moreover, improving nano-sensor technology could revolutionize crop health and soil conditions monitoring. Nano-sensors incorporated in agricultural fields could give real-time data on soil moisture, nutrient status, and incidence of pests or diseases. Subramanian, et al. [93] indicated how this data could be used to optimize irrigation, fertilization, and pest control in farming, making it more precise and resourceful.

The other promising way forward is the development of nanoparticle-mediated gene-editing tools, including CRISPR-Cas systems. Such applications may be used for engineering crop pest and disease resistance at the genetic level, which could be more durable and stable than conventional chemical treatments [94]. Another line of development is in the area of biodegradable nanoparticles being developed in line with enhancing fears on sustainability in agriculture. These nanoparticles produce the same advantages as the non-degradable nanoparticle forms. Still, at the end of their useful life, they break down into inert products with no further contribution to the proliferation of nanomaterials in the environment [95].

## 8. Conclusions

This study underscores the promising role of nanoparticles in advancing sustainable agricultural practices, particularly through enhanced pest control and improved plant resilience. By analyzing multiple nanoparticle types—such as silver, zinc oxide, and silica—this work illustrates how these materials impact pest and plant physiology through specific mechanisms. For example, silver and zinc oxide nanoparticles disrupt insect biological processes by inducing oxidative stress, while silica strengthens plant structural defenses, effectively enhancing pest deterrence. These findings suggest that nanoparticles could offer environmentally friendly alternatives to conventional pesticides, contributing to reduced chemical usage in agriculture. However, while nanoparticles offer these potential benefits, concerns regarding their long-term environmental impact persist. Issues such as bioaccumulation, persistence in soil and water, and effects on non-target organisms highlight the need for careful consideration of their ecological implications. Current regulatory frameworks remain insufficiently equipped to address these complex challenges, underscoring the need for standardized guidelines and interdisciplinary research to assess nanoparticles’ environmental and health impacts over time. This study lays a foundation for integrating nanoparticles into agricultural systems, encouraging further research focused on balancing the technological benefits with safety considerations. Addressing these research gaps will be essential for responsibly harnessing the potential of nanoparticles, ensuring they contribute effectively and sustainably to modern agricultural practices.

## Figures and Tables

**Table 1:** Effects of different nanoparticles on various crop plants and their molecular responses.

Nanoparticle	Positive Effects	Affected Pathways	Specific Genes	References
Silver nanoparticles	Enhance plant growth and pest resistance	Defense-related pathways	ROS, oxidative stress genes	[61,64]
Zinc oxide nanoparticles	Boost salicylic acid pathway, increase nutrient uptake	Salicylic acid pathway	SA-related genes	[62]
Silica nanoparticles	Strengthen cell walls, enhance tolerance to stresses	Jasmonic acid pathway	JA-related genes	[60,63]
Copper nanoparticles	Strong antimicrobial properties, reproductive disruption in pests	Hormone regulation pathways	Reproductive and developmental genes	[65,66]
Titanium dioxide nanoparticles	Enhance photosynthesis and biomass production	Photosynthetic pathways	Chlorophyll photoreactivity genes	[40]
Iron oxide nanoparticles	Improve nutrient uptake	Nutrient uptake pathways	Iron assimilation genes	[59]

**Table 2:** Effects of different nanoparticles on various insects and their molecular responses.

Nanoparticle	Negative Effects	Affected Pathways	Specific Genes	References
Silver nanoparticles	Lowered survival, disturbed reproduction cycles	Oxidative stress pathways	Detoxification genes, ROS	[43,56]
Copper nanoparticles	Impair embryonic development, decrease egg hatchability	Hormone regulation pathways	Reproductive genes	[65,66]
Zinc oxide nanoparticles	Alter movement patterns, reduce feeding efficiency	Nutrient metabolism pathways	Digestive enzymes	[71,78]
Silica nanoparticles	Disrupt digestive enzyme function, decrease nutrient absorption	Nutrient metabolism pathways	Digestive enzymes	[58,79]
Titanium dioxide nanoparticles	Induce oxidative stress, impair energy metabolism	Oxidative stress pathways	Stress-related proteins, energy metabolism genes	[81,82]
Iron oxide nanoparticles	Affects detoxification systems, reduces reproductive success	Detoxification pathways	Cytochrome P450, GSTs, carboxylesterases	[83]

The effects and molecular responses listed here are based on extrapolations from other pest species, as limited studies specifically address the impact of nanoparticles on *T. absoluta*. This extrapolative approach offers insights into potential mechanisms but requires direct validation in *T. absoluta*.

## Data Availability

No new data were created or analyzed in this study. Data sharing is not applicable to this article.
